# MSIonization: A Machine Learning Tool for Ionization Mode Prediction of Small Molecules

**DOI:** 10.1021/acs.analchem.6c01937

**Published:** 2026-09-16

**Authors:** Yatendra Singh, Bakhtyar Sepehri, Zeyad Ibrahim, Robert J. Doerksen, Sixue Chen

**Affiliations:** † Department of Biology, 8083 University of Mississippi, Oxford, Mississippi 38677, United States; ‡ Department of BioMolecular Sciences, 8083 University of Mississippi, Oxford, Mississippi 38677, United States

## Abstract

Selection of the optimal ionization mode to use–positive or negative–is a critical step in liquid chromatography–mass spectrometry (LC-MS) analysis of small molecules acquired using electrospray ionization (ESI) or atmospheric pressure chemical ionization (APCI). However, determining which ionization mode provides higher ionization efficiency usually relies on empirical knowledge or trial-and-error, particularly for large and chemically diverse compound libraries, which is time-consuming and resource-intensive, representing a major barrier in MS analysis. To address this challenge, we developed MSIonization, a machine learning (ML)-driven tool that predicts the preferred ionization mode (positive or negative) of small molecules. It performs binary classification using molecular structure (SMILES) as input to deliver rapid, scalable predictions accompanied by probability scores and applicability domain assessments, thereby facilitating selection of the preferred ionization mode and improving experimental planning. It offers a user-friendly, offline ML model with a graphical user interface (GUI) that streamlines the MS workflow and minimizes trial-and-error. MSIonization provides a practical decision-support framework that complements chemical intuition and supports experimental planning across a wide range of scientific domains. Looking ahead, it holds significant potential to integrate into AI-driven MS pipelines to predict the ionization mode of chemically diverse small molecules at a large scale to support research across several scientific domains. The package is available in the PyPI repository (https://pypi.org/project/MSIonization/) and can be installed using 'pip install MSIonization' command.

## Graphical abstract

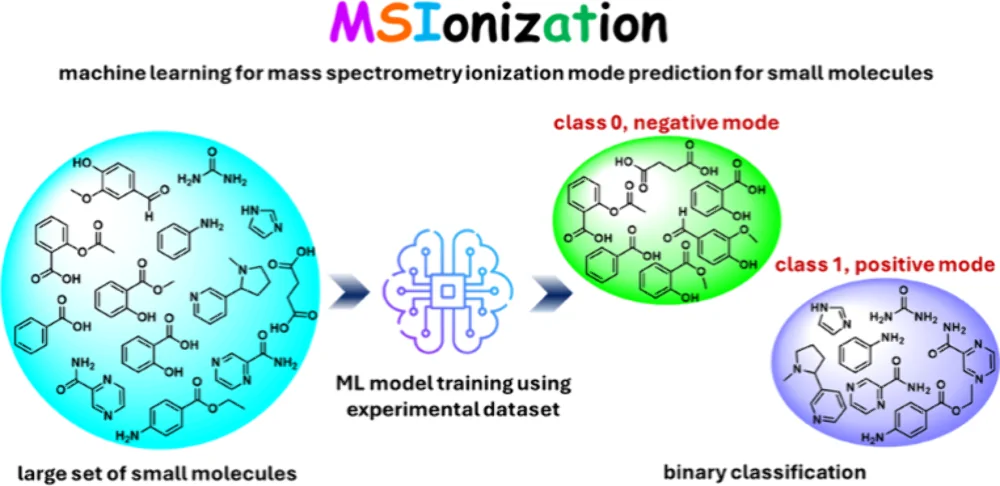

## Introduction

Mass spectrometry (MS) is a pivotal analytical technique for identifying and quantifying small molecules in complex samples.^1–3^ Tandem mass spectrometry (MS/MS) yields a unique fragmentation pattern of the analyzed compound, offering valuable insights into its composition and structural information.^4,5^ Liquid chromatography (LC) coupled with MS/MS (LC-MS/MS) enables sensitive and high-throughput analysis across metabolomics,^6,7^ drug discovery,^4,8^ forensic studies, and environmental sciences.^9^ Despite its widespread use, experimental performance in LC-MS/MS critically depends on selecting an appropriate ionization mode, typically positive or negative, which strongly influences sensitivity, reproducibility, and overall detection efficiency.

Small molecules (typically <1500 Da)^10^ exhibit diverse ionization behaviors depending on their chemical structures, functional groups, and experimental conditions. Although ionization efficiency has been extensively studied to understand factors contributing to their ionization process,^11–13^ and several ionization efficiency prediction models have been proposed,^13–16^ selecting the preferred ionization mode for a given molecule remains challenging, particularly when analyzing large and chemically diverse compound sets. In practice, ionization mode selection often relies on empirical rules or trial-and-error experimentation, which becomes impractical in high-throughput workflows and large-scale screening studies.

In many LC-MS workflows, data can be acquired in both positive and negative ionization modes to maximize molecular coverage. However, dual-polarity acquisition approximately doubles instrument acquisition time and solvent consumption, increasing analytical cost while reducing sample throughput. These limitations become particularly important in high-throughput applications involving thousands of compounds, such as targeted method development, suspect screening, metabolomics, pharmaceutical research and environmental monitoring. For example, environmental monitoring routinely involves the analysis of chemically diverse contaminants, including pesticides, fungicides, industrial chemicals, and pharmaceuticals.^17^ Consequently, a computational tool capable of rapidly prioritizing the preferred ionization mode can substantially improve analytical efficiency by reducing unnecessary experimental optimization and streamlining large-scale LC-MS workflows.

Machine learning (ML) algorithms have emerged as promising tools for tackling these challenges by learning complex relationships from high-quality and vast datasets of experimental data.^12–14^ Recent studies have demonstrated the feasibility of predicting LC-MS detectability through capturing the physicochemical properties of the analytes^18^ and proposed an artificial neural network model for ionization mode prediction.^19^ While these models provide important advances in predicting ionization mode and overall LC-MS detectability, they remain restricted to binary classification of environmental pollutants. To our knowledge, there is currently no broadly applicable ML tool that predicts the preferred positive or negative ionization mode while simultaneously providing prediction probabilities and applicability domain assessments.

Here, we present MSIonization, an ML tool developed to predict the preferred ionization mode (positive or negative) of small molecules directly from their molecular structures (SMILES). This ML model was trained on curated experimental data comprising 9,168 compounds acquired in-house and obtained from mzCloud. In addition to predicting the preferred ionization mode, MSIonization provides probability scores and applicability domain assessments to assist users in evaluating prediction confidence. Implemented as an easy-to-use Python package with both command-line and graphical user interfaces (GUI), MSIonization enables rapid, scalable, and offline prediction of ionization mode, facilitating experimental planning and supporting applications in metabolomics, pharmaceutical analysis, forensic science, natural products research, and environmental studies.

## Experimental Section

### Data Set Preparation

Two datasets, the mzCloud dataset and the natural products dataset, were prepared for model development. The mzCloud dataset contains experimental data acquired using electrospray ionization (ESI), nanoelectrospray ionization (nano-ESI), and atmospheric pressure chemical ionization (APCI), each operated in positive and negative ionization modes. The natural products dataset generated in the Chen lab was acquired exclusively using ESI in both positive and negative ionization modes.

The LC-MS data were downloaded from the mzCloud database (accessed December 10, 2024),^20^ a curated database for high- and low-resolution MS^n^ spectra (Figure 1). It encompasses 17 classes, including counterfeit/therapeutic drugs, abuse/illegal drugs, endogenous metabolites, excipients/additives/colorants, extractable/leachable, illegal additives, industrial chemicals, natural products/medicines, natural toxins, perfluorinated hydrocarbons, personal care products/cosmetics, pesticides/herbicides, sports doping drugs, steroids/vitamins/hormones, textile chemicals/auxiliary/dyes, therapeutics/prescription drugs, and veterinary medicines. To ensure data quality, redundant entries across chemical classes were removed. Each compound’s name, molecular formula, SMILES, and chemical class information were saved in an Excel sheet. Compound names were subsequently queried in the mzCloud database to retrieve ionization mode and ionization efficiency information. In total, ionization data for 8099 molecules were collected and designated as the mzCloud dataset (Table S1).

**Figure 1:**
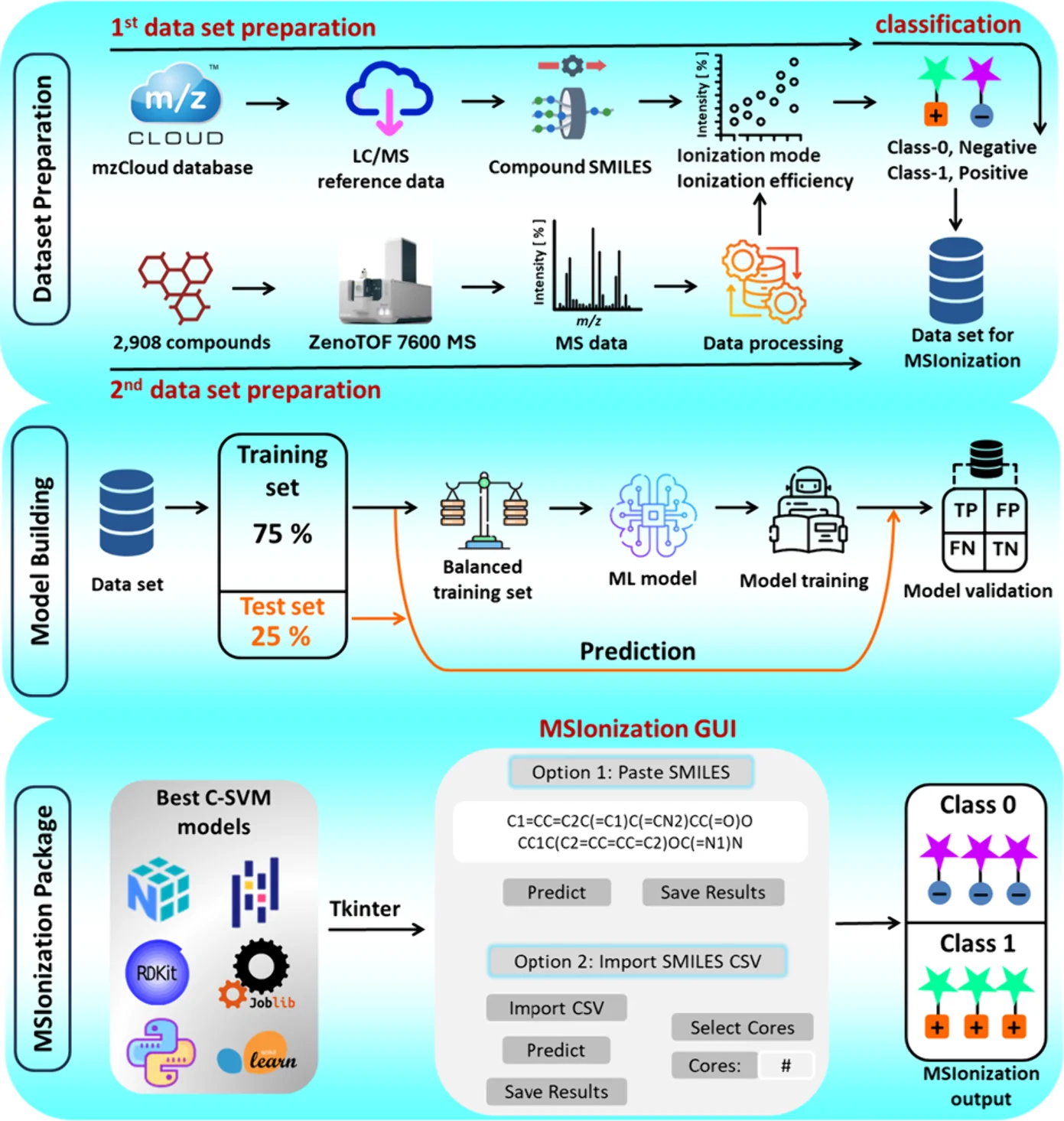
Schematic workflow for developing the MSIonization package. The LC-MS reference data were collected from mzCloud and supplemented with experimental data acquired on the ZenoTOF 7600 MS. These datasets were processed to extract information on ionization mode and ionization efficiency. Each compound was subsequently labeled as Class 0 for negative ionization mode or Class 1 for positive ionization mode, forming the basis of the MSIonization model building dataset. The dataset was split into a training set and an external test set. The training set was balanced to include approximately equal numbers of Class 0 and Class 1 molecules and used to train various ML models. The best-performing model, a C-SVM model, was selected for use in the MSIonization package. The software calculates all MACCS key fingerprints for the molecules and uses the best C-SVM model for predicting the ionization mode of the molecules.

The natural products dataset was prepared in the Chen lab (Figure 1). A natural products library comprising 2802 compounds was purchased from MedChemExpress, and an additional 106 compounds were included from the Chen lab inventory. Samples were prepared by combining 16 compounds at a time, each at a concentration of 1 ng/μL, in a 50:50 (v/v) mixture of acetonitrile and water containing 0.1% formic acid.^21^ Analyses were performed on Acquity UPLC-ZenoTOF 7600 MS (Sciex, ON, Canada) equipped with an OptiFlow TurboV ion source. The separation was achieved on nanoEase M/Z Symmetry C18 (5 μm, 300 μm i.d. × 50 mm, 100 Å) column at a flow rate of 25 μL/min. The total run time was 7 min, including a 4 min linear gradient of water and acetonitrile containing 0.1% formic acid.^21^ Data were acquired using both positive and negative modes to ensure comprehensive ionization data for each molecule. The resulting raw data were manually examined using a peak-picking approach via Sciex OS Explorer to extract ionization mode and ionization efficiency information. This analysis yielded ionization information for 2908 molecules (Table S2).

Each dataset was compiled in an Excel sheet, containing compound name, chemical formula, SMILES notation, ionization mode, and ionization efficiency. The two datasets (Tables S1 and S2) were merged and subjected to further curation to improve data quality. Redundant entries were removed, including 245 duplicate molecules, 190 adduct ions, 28 metal-containing molecules, seven silicon-containing molecules, and 1369 molecules with a molecular weight exceeding 800 Da. This filtering process resulted in a curated dataset of 9168 unique small molecules (Table S3), which was used for model training and validation.

For binary classification model development, each molecule in the curated dataset was assigned to one of two classes based on its dominant ionization mode. Molecules that preferentially ionized in negative mode were labeled as Class 0, while those that preferentially ionized in positive mode were labeled as Class 1. Molecules that were ionizable in both positive and negative modes, for which the class was determined by the mode with higher ionization efficiency, were also included in training sets. Molecules detected exclusively in one ionization mode were assigned directly to that corresponding class. This approach ensures that the model can learn to predict the preferred ionization mode while retaining molecules that are detectable in either mode or both modes, thereby maximizing the chemical diversity of the training dataset.

### Chemical Diversity Analysis

Principal component analysis (PCA)^22^ was performed using the singular value decomposition (SVD) algorithm on the molecular access system (MACCS) keys fingerprint space in the scikit-learn package. The resulting score plot (Figure S1A) shows no distinct or well-defined groups of molecules broadly distributed across the score space. The widespread occurrence along both principal components suggests that the chemical structures comprise diverse combinations of substructures, with no single dominant scaffold. This demonstrates that the dataset includes a diverse set of chemical structures. Consistent with this observation, Murcko scaffold analysis performed using RDKit identified 3101 unique scaffolds, further supporting the substantial structural heterogeneity of molecules.

To further assess the chemical diversity, pairwise Tanimoto similarity was calculated using Morgan fingerprints (radius = 2, fingerprint length = 1024 bits). The mean, median, and standard deviation of the Tanimoto similarity coefficients were 0.1054, 0.0983, and 0.0553, respectively, indicating low overall structural similarity among the molecules. The pairwise similarity matrix and the corresponding frequency distribution are presented in Figure S1B,C, respectively. Duplicate pairwise similarity values were removed by considering only the upper triangular portion of the similarity matrix. The majority of pairwise similarities were below 0.2, highlighting the high structural diversity of the molecules in the dataset.

Structural diversity was further assessed using pairwise Levenshtein distances and similarities calculated from canonical SMILES strings. The pairwise Levenshtein distance matrix, similarity matrix, and distance distribution are shown in Figure S1D–F, respectively. The mean and median values for the Levenshtein distance were 38.59 and 35, respectively and for Levenshtein similarity are 0.4256, and 0.4294, respectively. Standard deviation of Levenshtein distances and similarities are 16.09 and 0.1174, respectively. The Levenshtein distance exceeded 20 for more than 80% of molecule pairs. Collectively, the PCA, Murcko scaffold analysis, Tanimoto similarity, and Levenshtein distance analyses demonstrate that we have used a highly diverse dataset for developing the ML model.

### Molecular Property Distributions

Distributions of key molecular properties, including molecular weight, logP, and numbers of carbon, nitrogen, and oxygen atoms, are shown in Figure S2A,B. These molecular properties were calculated using RDKit package. Molecular weights ranged from 59 to 800 Da, with most molecules between 150 and 450 Da. The mean and median molecular weights are 333 and 323 Da, respectively. The maximum numbers of carbon, oxygen, and nitrogen atoms observed in any single molecule are 45, 24, and 10, respectively, with most molecules containing between 6 and 26 carbon atoms. Nitrogen-free molecules were substantially more abundant than oxygen-free molecules. In general, most molecules contain 1–6 oxygen atoms, while more than 4000 molecules contain no nitrogen atoms. Median values for carbon, nitrogen, and oxygen atoms are 17, 1, and 4, respectively. LogP values range from −9.7488 to 14.7569, with most molecules exhibiting logP values between 0 and 5. The mean and median logP values are 2.226 and 2.367, respectively.

### Program Packages

Spyder (v6.0.3)^23^ was used as an integrated development environment for developing and testing all necessary code written in the Python programming language (v3.9.21).^24^ The RDKit package (v2022.09.5)^25^ was used to calculate Murcko scaffold analysis,^26^ Morgan fingerprints,^27^ Tanimoto coefficient,^28^ and all 166 MACCS key fingerprints.^29^ The scikit-learn package (v1.6.0)^30^ was used to build ML models and perform statistical analyses and PCA^22^ using the SVD algorithm. The matplotlib package (v3.9.4)^31^ was used to generate plots. The sklearn-genetic-opt package (v0.11.1)^32^ was used to optimize hyperparameters of each ML model and select informative variables using a genetic algorithm. We wrote a Python code for calculating leverage values to define the applicability domain of each model. The Levenshtein package (version 0.27.3)^33^ was used to calculate the Levenshtein distance and similarity of molecules available in the dataset. The SHAP package^34^ was used for interpreting the contribution of the input variables in the building of the best ML model.

### Comparison with Other Datasets

To further assess the structural diversity and applicability domain of the training dataset, we compared it with two large external chemical databases: the NPAtlas database of natural products (36454 molecules) and the NORMAN SusDat database (89092 molecules).

For the NPAtlas database, the mean, median and standard deviation for pairwise Tanimoto similarity were 0.1201, 0.1162, and 0.0446, respectively. The corresponding mean, median, and standard deviation of the Levenshtein similarity were 0.4465, 0.4559, and 0.1171, respectively, whereas those for the Levenshtein distance were 76.0600, 63.0000, and 49.2488, respectively. The distributions of pairwise Tanimoto similarities and Levenshtein distances are shown in Figure S3.

For the NORMAN SusDat database, the mean, median and standard deviation of pairwise Tanimoto similarities were 0.0868, 0.0800, and 0.0560, respectively. The corresponding values for the Levenshtein similarity were 0.3761, 0.3783, and 0.1370, whereas those for the Levenshtein distance were 40.7800, 32.0000, and 38.9500, respectively. The distributions of pairwise Tanimoto similarities and Levenshtein distances are presented in Figure S4.

The distributions of Tanimoto similarities and Levenshtein distances for all datasets show a vast majority of calculated pairwise Tanimoto similarities and Levenshtein distances are smaller than 0.2 and 100, respectively, indicating extensive structural diversity. Collectively, these analyses indicate that the training dataset spans a broad chemical space comparable in diversity to those represented by the NPAtlas and NORMAN databases, supporting the general applicability of the developed ML models across structurally diverse small molecules.

### Machine Learning Model Selection

To develop classification models, different ML algorithms in the scikit-learn^30^ package including AdaBoost,^35^ Bernoulli Naïve Bayes,^36^ C-support vector machine (C-SVM),^37^ decision tree,^38^ extra tree,^39^ random forest,^40^ extra trees,^39^ Gaussian Naïve Bayes,^41^ Gaussian process,^42^ gradient boosting,^43^ histogram-based gradient boosting,^44^ k-nearest neighbors,^45^ linear support vector machine (Linear-SVM),^37^ logistic regression,^46^ multi-layer perceptron,^47^ and Nu-support vector machine (Nu-SVM)^37^ were used. Table S4 shows the list of optimized hyperparameters for each ML model along with their corresponding search spaces.

Stratified splitting^48^ of the dataset was carried out using scikit-learn, placing 25% of the molecules from each class in a test set for external validation. The remaining 75% of the molecules from each class were used for building the ML models (Figure 1). A genetic algorithm was used to find the best values for each hyperparameter used in each model to obtain an optimally predictive model. Both the number of generations and the population size in each generation were set to 10.

For finding the best hyperparameter values using the genetic algorithm, the weighted average of calculated F1 scores^49^ for each class of molecules in a stratified k-fold cross-validation (CV) test^45^ was considered as the evaluation metric. This metric was maximized during the optimization process. The best model has hyperparameter values that provide the maximum weighted average of F1 scores. The weighted average F1 score is calculated as

1 $$F1_{\mathrm{weighted avg}}=x_{\mathrm{Class0}}F1_{\mathrm{Class0}}+x_{\mathrm{Class1}}F1_{\mathrm{Class1}}$$

where *x*_Class0_ and *x*_Class1_ are the fractions of molecules with label 0 and label 1, respectively.

In a k-fold CV test, the training set is divided randomly into *k* subgroups. We used stratified k-fold CV testing, which uses the stratified splitting of samples by ionization mode class in the training set. This approach allows us to have the same distribution of samples belonging to each class in each subgroup. We used *k* = 5, so 20% of each class of molecules was put in each subgroup. Each time, four subgroups were used to build a model, and a prediction was made for molecules in the omitted subgroup. This process was repeated for each of the subgroups until the prediction had been performed for all the molecules in the training set.

It is important to balance the dataset for each class in the training set to ensure that we can have models with high predictive power for each class. Since Class 1 had many more molecules than Class 0, we used the neighborhood cleaning rule algorithm implemented in the imbalanced-learn package (v0.12.4)^50^ to remove noisy samples from Class 1 in the training set. Additionally, the voting classifier method^51^ was used to build a voting model of class prediction, in which the best obtained model by each ML method is used for predicting each molecule in both the training and test sets, and the final predicted class for each molecule is determined by majority rule voting, including results from all the ML methods.

### Calculated Metrics for Model Validation

For validating models, the accuracy was calculated for both the training and test sets:

2 $$\mathrm{accuracy}=\frac{\mathrm{TP}+\mathrm{TN}}{\mathrm{TP}+\mathrm{FN}+\mathrm{TN}+\mathrm{FP}}$$

where TP, TN, FP, and FN stand for true positive (the model correctly predicts that a molecule is Class 1), true negative (the model correctly predicts that a molecule is Class 0), false positive (the model incorrectly predicts that a molecule is Class 1), and false negative (the model incorrectly predicts that a molecule is Class 0), respectively.

To check the predictive power of the model for each class (Class 0 and Class 1) of molecules in both the training and test sets, we calculated precision, recall, and F1 scores for each class of molecules in both sets as

3 $${\mathrm{Precision}}_{\mathrm{Class1}}=\frac{\mathrm{TP}}{\mathrm{TP}+\mathrm{FP}}$$

4 $${\mathrm{Precision}}_{\mathrm{Class0}}=\frac{\mathrm{TN}}{\mathrm{TN}+\mathrm{FN}}$$

5 $${\mathrm{Recall}}_{\mathrm{Class1}}=\frac{\mathrm{TP}}{\mathrm{TP}+\mathrm{FN}}$$

6 $${\mathrm{Recall}}_{\mathrm{Class0}}=\frac{\mathrm{TN}}{\mathrm{TN}+\mathrm{FP}}$$

7 $${F1}_{\mathrm{Class1}}=\frac{2\mathrm{TP}}{2\mathrm{TP}+\mathrm{FP}+\mathrm{FN}}$$

8 $${F1}_{\mathrm{Class0}}=\frac{2\mathrm{TN}}{2\mathrm{TN}+\mathrm{FP}+\mathrm{FN}}$$

### Applicability Domain of Models

We used the leverage method to define the applicability domain of the developed model. The leverage was calculated for each molecule as

9 $$h_{i}=x_{i}{\left(X^{T}X\right)}^{-1}x_{i}^{T}$$

In eq 9, *h*_*i*_ is the leverage value for molecule *i*, *x*_*i*_ is the fingerprint vector for molecule *i*, and *X* is the fingerprint matrix for all molecules in the training set. The critical leverage was calculated as

10 $$\mathrm{Critical leverage}=\frac{\left(3\left(p+1\right)\right)}{n}$$

where *p* is the number of fingerprints (166 MACCS key fingerprints) used for developing the model, and *n* is the number of molecules in the training set. If the calculated leverage for a molecule is lower than the critical leverage, the predicted ionization mode for that molecule is considered reliable. However, leverage values exceeding the critical threshold indicate unreliable predictions.

## Results and Discussion

### Pipeline of MSIonization

The pipeline for MSIonization comprises (a) dataset preparation, (b) model building, and (c) running the MSIonization package, as depicted in Figure 1 and described in detail in the Experimental Section.

For the dataset preparation, LC-MS reference data were downloaded from the mzCloud, and natural products data were acquired in the lab (Tables S1 and S2). These data were processed to extract information on ionization mode (if positive, the molecule is placed in Class 1, or, if negative, the molecule is placed in Class 0) and experimentally observed ionization efficiency. After removing the duplicity and redundancy, in total, 9168 curated compounds were utilized for ML model training (Table S3). For the model building, the SMILES notations of the molecules were imported into Spyder, and 166 MACCS key fingerprints (bits 1 to 166) were calculated for each molecule using the RDKit package (Table S5).

For model building, the molecules were first randomly reordered, and then 25% of each class was allocated to the test set. After this step, we used two approaches. In the first approach, the training set was used both for finding the best hyperparameter values and for training the model. Since we have an imbalanced dataset, a second approach was applied to enhance the predictive power for molecules in Class 0. In this method, under-sampling was performed using the neighborhood cleaning rule algorithm, resulting in a refined training set. This new training set was approximately balanced, containing 5234 molecules, with 2947 belonging to Class 1 and 2287 molecules to Class 0. After finding the best values of hyperparameters for this new training set by using a genetic algorithm,^52^ models were trained using this new balanced training set, and ultimately, prediction was made for all molecules in the original training set and test set. Finally, we calculated statistical parameters for both classes of molecules in the training and test sets.

For the MSIonization package, the best C-SVM model was selected. The package uses the RDKit software to compute MACCS key fingerprints from the SMILES notation of molecules. Additionally, numpy, pandas, and scikit-learn tools are necessary to make the prediction, starting from the SMILES notation. The joblib software is required to load the pre-trained model. To make MSIonization easier to use, we built a GUI to provide two options: first, to paste a list of SMILES strings, and second, to import a SMILES CSV, called batch prediction.

### Building Models Using an Unbalanced Training Set

In the initial modeling attempt, prediction models were trained using the original, unbalanced training set without applying any under-sampling strategy. Across different ML algorithms, the weighted-average F1 score obtained from k-fold cross-validation ranged from 0.31 to 0.78, while test set accuracy ranged from 0.33 to 0.77 (Figure 2A, top panel). However, for imbalanced datasets, accuracy is not a reliable metric for evaluating the predictive power of the model for each molecular class. Therefore, precision, recall, and F1 scores were calculated separately for each molecular class in both the training and test sets (Figure 2B, top panel). Precision and F1 scores are affected by the imbalance in the number of molecules in each class, whereas recall remains unaffected.

**Figure 2:**
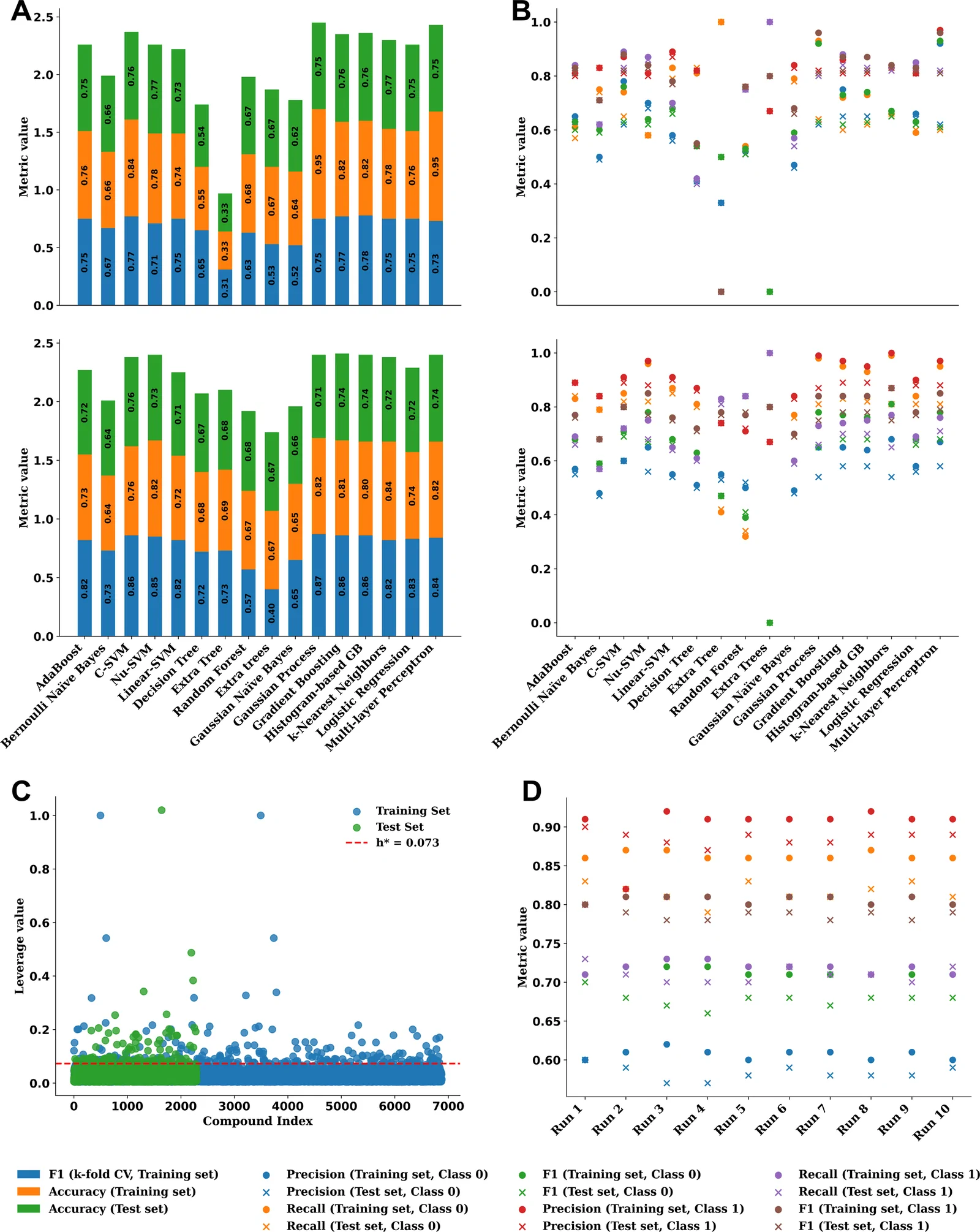
Performance evaluation of the developed machine learning models. (A, B) Statistical performance metrics for models trained on the original (top) and balanced (bottom) training sets. (C) Leverage values for molecules in the training and test sets of the C-SVM model, illustrating the applicability domain; h* represents the critical leverage, and the dashed line shows the critical value. (D) Statistical performance metrics across 10 random splits of the dataset for the C-SVM model.

As shown in Figure 2B (top panel), recall values were acceptable for both Class 0 and Class 1 across the decision tree, extra tree, random forest, and extra trees models. Bernoulli Naïve Bayes, Linear-SVC, and Gaussian Naïve Bayes exhibit high recall for Class 0 in the test set, but their predictive performance for Class 1 is low. Moreover, Bernoulli Naïve Bayes and Gaussian Naïve Bayes show an unacceptably low precision for Class 0 in the test set. For most other models, recall values for Class 0 in the test sets of other models indicate low predictive power. Generally, both F1 scores and precision values are low for Class 0 in the test set. In contrast, AdaBoost, C-SVM, Nu-SVM, Gaussian process, gradient boosting, histogram-based gradient boosting, k-nearest neighbors, logistic regression, and multilayer perceptron models show strong predictive performance for Class 1 in the test set, with high precision, recall, and F1 scores. However, for Class 0, these models achieve only moderate performance.

To further improve predictive performance for Class 0, a voting classifier model was built that used the best-trained model for all 16 ML algorithms. In the test set, this ensemble model achieved precision, recall, and F1 scores of 0.64 for Class 0 and 0.82 for Class 1, indicating no substantial improvement in predictive power for Class 0 compared with individual models.

### Building Models Using the Under Sampling Approach

To improve predictive performance for Class 0, the dataset was split, and the training set was balanced using the neighborhood cleaning rule under-sampling algorithm. Out of 9168 molecules, 6876 were assigned to the training set, comprising 4589 molecules for Class 1 and 2287 molecules for Class 0. After under-sampling, the refined training set contained 5234 molecules, including 2947 molecules for Class 1 and 2287 molecules for Class 0. This new training set was used to train models.

Figure 2A and B (bottom panels) show the calculated statistical parameters of models trained, which were applied to the original training and test sets. Compared with the models trained on the unbalanced dataset, these results show a substantial improvement in predictive performance for Class 0 in both the training and test sets. Notably, C-SVM, Nu-SVM, gradient boosting, histogram-based gradient boosting, logistic regression, and multilayer perceptron models exhibited marked improvements. Among these, the C-SVM model demonstrated the highest overall predictive performance for both Class 0 and Class 1 in the test set (Figure 2B, bottom panel). Again, we created a voting classifier for these trained models. Precision, recall, and F1 scores for Class 0 were 0.58, 0.82, and 0.68, respectively, and for Class 1, they were 0.89, 0.70, and 0.78, respectively in the test set of this model. These results indicate that the C-SVM model has slightly higher predictive power for Class 1 in the test set than the voting model.

Based on these comparisons, the C-SVM model was selected as the final model and implemented in a Python package. The *C*, kernel, gamma, and probability parameters were set to 0.756734, rbf, scale, and True, respectively, for this C-SVM model. Using only molecular fingerprints, the model correctly predicts the preferred ionization mode for 76% of molecules, indicating that molecular structure is an important determinant of ionization mode. Prediction uncertainty is reflected in the Class probabilities, with values approaching 0.5 indicating ambiguity in which neither ionization mode is strongly favored. Such compounds warrant acquisition in both positive and negative ionization modes. However, other experimental factors, such as solvent composition, pH, and adduct formation, were not included in the model, so prediction accuracy is not expected to reach 100%.

### Applicability Domain of C-SVM Model

The critical leverage value for the C-SVM model was 0.073. As shown in Figure 2C, most of the molecules fall within the model’s applicability domain, indicating reliable predictions. Approximately 97% of molecules in the training set and 96% of molecules in the test set were within this domain. Although the model can predict ionization mode for compounds with molecular weights exceeding 800 Da, prediction reliability in this higher mass range is uncertain, as the training data were limited to molecules with molecular weights between 59 and 800 Da.

### Effects of Data Splitting and Variable Selection on C-SVM Performance

To investigate the effect of dataset splitting on the C-SVM model performances, the dataset was randomly split ten times. For each split, the neighborhood cleaning rule algorithm was applied to balance the training set, and the resulting dataset was used to train the C-SVM model. Hyperparameter optimization was not repeated at this stage; instead, the optimized hyperparameters from the best-performing C-SVM model were reused. Precision, recall, and F1 scores for both molecular classes in the training and test sets across the ten splits are shown in Figure 2D. Across all ten runs, no significant variation in predictive performance was observed for either class in either the training or test sets. This demonstrates that splitting the dataset does not influence the model’s predictive power.

To assess the effect of variable selection, a genetic algorithm^53^ was employed to identify informative molecular fingerprint descriptors. Of the 166 MACCS key fingerprints, 91 were selected as informative variables. Using these fingerprints, a balanced training set was generated via under-sampling, followed by variable selection and hyperparameter optimization to train a new C-SVM model. In the test set, precision, recall, and F1 scores for Class 0 were 0.60, 0.82, and 0.69, respectively, while for Class 1 they were 0.89, 0.72, and 0.80. These values are comparable to those obtained using all 166 fingerprints. Because variable selection did not lead to a meaningful improvement in predictive performance, all 166 MACCS key fingerprints and the balanced training set were retained for development of the MSIonization package.

### Interpretation of Input Variables in C-SVM Model

SHAP analysis identified chemically meaningful MACCS fingerprint keys that govern ionization mode preference. Fingerprints associated with acidic and oxygen-rich functional groups predominantly led to predictions of negative ionization mode, consistent with the ability of such functional groups to undergo deprotonation and stabilize negative charge. Conversely, fingerprints representing nitrogen-containing functional groups increased the probability of prediction of positive ionization mode because of their high proton affinity. These results demonstrate that the C-SVM model captures chemically meaningful structure–ionization relationships rather than relying solely on statistical correlations. Figure S5 includes beeswarm and bar plots for the 19 most informative MACCS fingerprints. These plots provide a global explanation for feature importance. The waterfall plots in Figure S6 provide local interpretation of feature importance for molecules M1, M35, M371, and M2188. Based on these plots, we have explained feature importance for the C-SVM model in Supporting Information, text 1.

### The MSIonization Package

The MSIonization package employs the C-SVM model to predict the dominant ionization mode of molecules, requiring only SMILES notation as input. For each molecule, all 166 MACCS key fingerprints are automatically calculated and passed to the trained C-SVM model for prediction. To make a prediction, the *predict_ionization* function from the MSIonization package must be loaded. The output is a dictionary that provides the predicted class, a probability score, and an applicability domain assessment. The probability score indicates the likelihood that the molecule belongs to Class 0 or Class 1. The applicability domain assessment specifies whether the molecule falls within the applicability domain of the trained C-SVM model.

To facilitate batch processing, a Python script named *MSIonization_script.py* was developed that accepts a comma-delimited CSV file (SMILES.csv) containing multiple SMILES strings. The file must include a header “SMILES” in the first row and each subsequent row containing a single SMILES string. To use the script, place both the script and the CSV file in the same directory. To run this script, open a terminal and run the following command: python3 *MSIonization_script.py*. Upon execution, the script automatically detects the maximum number of available CPU cores and prompts the user to select the number of cores to be used for computation. The selected number of cores is then utilized at full capacity during prediction.

In addition to the command-line interface, a GUI is available and can be launched by typing MSIonization-GUI in the terminal. The GUI consists of two primary panels for prediction (Figure 1). The first panel is for pasting a list of SMILES strings directly, and the other is for importing a CSV file (SMILES.csv) containing a SMILES column. Each panel includes a ‘Predict’ button to start the calculations and a ‘Save Results’ button to export the results as a CSV file. A third panel displays the automatically detected maximum number of available CPU cores and allows users to adjust the number of cores used for computation.

To benchmark performance, MSIonization was used to predict ionization modes for all 9168 molecules in the curated dataset on a system equipped with an Intel Core i9-10900X CPU@3.70 GHz running Ubuntu 20.04.4 LTS. The total execution time was 1144.78 seconds, of which 1144.66 seconds were spent on prediction and 0.12 seconds on writing results to a CSV file. The prediction speed on this machine was 8.01 molecules per second.

The MSIonization represents a data-driven approach in binary classification of small molecules for their ionization mode, predicting whether a molecule is most likely to ionize in the positive or negative mode. The package is designed for large-scale analyses and can process hundreds of thousands of molecules in a single run. It is designed to be user-friendly and easy to install. It can run on Linux, Windows, and macOS and does not require an internet connection or advanced computational expertise. Supporting text 2 provides a detailed tutorial for installing and running this package, and it is available at https://www.moleculardetective.org/Links.html

### Large Chemical Space

To assess scalability and practical utility, MSIonization was applied to two large and chemically distinct databases. NPAtlas^26^ contains 36453 microbially derived natural products, and the NORMAN SusDat^54^ database contains 89092 environmental contaminants. Together, these datasets span broad structural and functional group diversity relevant to metabolomics and environmental screening. Batch prediction was performed on an Intel Core i9-10900X CPU at 3.70 GHz with 20 cores under Ubuntu. NPAtlas predictions finished in 4541.61 s (∼75.7 min) at 8.03 molecules/s, while NORMAN SusDat predictions took 11443.50 s (∼190.7 min) at 7.79 molecules/s.

For NPAtlas,^26^ 12518 molecules (34.34%) were predicted in positive mode (Class 1) and 23935 (65.65%) in negative mode (Class 0). Outputs include Class 0 and Class 1 labels and probability scores that indicate the confidence level of each prediction, providing valuable quantitative guidance to users. For example, curvularide C is predicted as Class 0 with 5.1% probability and Class 1 with 94.9% probability (Table S6). Importantly, 35087 molecules (96.25%) fall within the model’s applicability domain, while 1366 (3.74%) are out-of-domain due to structural differences or underrepresentation in training data. The applicability domain is based on fingerprint similarity rather than molecular weight, so some high-mass compounds, e.g., polytheonamides A and B (5033 Da) are in-domain with predicted probabilities of 62.2% positive and 37.8% negative (Table S6), though such predictions should be interpreted cautiously.

Ionization mode preference is largely governed by well-established chemical principles, particularly that amine-containing molecules favor positive mode, whereas carboxylic acids, phenols, or sulfonates favor negative mode. For example, cochlearin H, which contains acidic and alcohol functional groups, strongly favors negative ionization mode with 98.47% probability, while tetrahydroindol 3, with an amine group, prefers positive mode at 94.99% probability (Table S6). While such behavior is chemically intuitive for many simple molecules, ionization mode selection becomes less clear for compounds containing multiple or chemically distinct functional groups, borderline pKa values, or increased structural complexity. Dual-mode or structurally complex molecules, such as companeramide A (1084.45 Da), which exhibits nearly equal probability scores of 47.53% for negative mode and 52.47% for positive mode, highlight ambiguity that is difficult to resolve using simple heuristic rules alone (Table S6). For such cases, MSIonization complements chemical intuition rather than replacing it.

For the NORMAN SusDat,^54^ 80335 molecules (90.17%) were within the domain, indicating reliable predictions for most of the dataset. Of these, 58891 (66.10%) were predicted to ionize in positive mode, while 30201 (33.89%) were in negative mode, with most compounds having mass between 100–800 Da and logP between 0 and 6. These results demonstrate that MSIonization enables large-scale, in silico pre-classification of environmentally relevant chemicals by ionization mode prediction into Class 1 or Class 0, supporting efficient experimental planning for environmental monitoring and toxicological studies.

Compared with Lowe et al.’s amenability model,^18^ which only classifies environmental compounds as amenable or unamenable to LC-ESI-MS detection, MSIonization predicts preferred ionization mode with probability scores and applicability domain assessment. This provides early-stage guidance, enabling informed ionization mode selection and reducing trial-and-error for method development across many scientific domains. Its implementation as a standalone Python package with a GUI enables scalable, offline, high-throughput prediction, supporting reproducible ionization mode assignment across a large chemical space.

### Future and Utility of MSIonization

Despite the promising results, MSIonization will undergo several improvements that provide clear directions for future development. First, the current model is restricted to predicting ionization mode for polar to moderately polar, non-volatile compounds analyzed using ESI, NSI, and APCI. It has not been trained on volatile compounds or data from other ionization techniques such as APPI or CI, nor does it account for matrix-dependent factors, including pH, acidity, or basicity. Expanding the training data to include additional ionization methods and more hydrophobic or volatile compounds would broaden the model’s applicability. Second, the training dataset is centered on molecules within the 50–800 Da range, whereas many MS applications involve higher-mass species, such as phosphatidylcholines, sphingomyelins, fatty acyl glycerides, and large polyketides (>800 Da), which are underrepresented or absent. Although MSIonization can generate predictions for compounds above 800 Da, performance in this higher mass regime remains uncertain. Future work will aim to expand the training set to include these underrepresented classes. Finally, the applicability domain of MSIonization is constrained by its chemical coverage. The training dataset encompasses 18 chemical classes covering the 9,168 molecules, which is much larger than that used in another recently reported model,^18^ but remains a relatively small dataset compared to the estimated one million natural metabolites.^27^

In the future, as more molecules that fall outside the chemical spaces represented in this report become available, the model’s utility can certainly be extended and enhanced. MSIonization is particularly useful for applications that require rapid or informed decision-making regarding ionization mode to guide LC-MS method development. For example, it can facilitate rapid screening and guide the selection of the preferred ionization mode for high-throughput metabolomics, suspect screening of environmental contaminants, and pharmaceutical analysis. By providing reliable predictions of ionization mode, it streamlines MS workflows across diverse fields.

This C-SVM model holds significant potential to integrate with AI-driven MS pipelines to predict the ionization mode of small molecules, thereby supporting research across a wide range of scientific disciplines. We have already demonstrated the utility of MSIonization in predicting ionization modes at a large scale across chemical space (Tables S6 and S7). Currently, widely used MS software platforms such as SCIEX OS, MassLynx, Xcalibur/FreeStyle, etc., are not configured with such an algorithm to predict ionization mode. Integrating MSIonization into these platforms could significantly streamline MS workflows and accelerate discovery by saving time and resources and reducing experimental trial-and-error. Additionally, the automatic ionization mode prediction makes MS accessible to many non-MS experts, such as clinical lab staff and physicians.

## Conclusion

In this study, we present MSIonization, an ML-driven tool developed to predict the ionization mode of small molecules. It achieves rapid, accurate, and scalable predictions by classifying molecules into positive and negative modes, providing associated probability scores and applicability domain assessments. Integration of MSIonization into MS analytical pipelines can streamline method development, reduce experimental trial-and-error, conserve time and resources, and improve reproducibility, thereby accelerating small molecule analysis. As such, MSIonization represents a meaningful advance in computational MS, offering a practical and robust decision-support framework for ionization mode selection. Future efforts will focus on further improving model performance and expanding the applicability domain by incorporating more chemically diverse training data, including underrepresented classes such as oligosaccharides, phosphatidylcholines, and sphingomyelins, as well as extending support to additional ionization techniques such as APPI and CI. Moreover, incorporating ionization efficiency estimates that account for physicochemical factors, including pH, acidity, and basicity, will enable a more realistic representation of molecular ionization behavior. Collectively, these developments will reduce out-of-domain predictions, enhance reliability for structurally novel compounds, and support robust ionization mode prediction across an even broader chemical space.

## Supplementary Material





## Data Availability

The supporting data for this study are available within the main manuscript and the Supplementary Information. A tutorial for installing and running the MSIonization package is available at https://www.moleculardetective.org/Links.html. The code for the MSIonization package can be accessed on GitHub at https://github.com/sepehribakhtyar/MSIonization.

## Notes

Principal components analysis, Tanimoto similarity and Levenshtein distance analyses of the training dataset; molecular property distributions; applicability domain assessment for the NPAtlas and NORMAN databases; waterfall plots illustrating representative prediction probabilities; and tutorial for installing and running the MSIonization package (PDF)
Supplementary Tables S1–S7 (XLSX)

## ∥

Department of Pharmacology, University of Virginia, Charlottesville, VA 22908, USA
