# Supplementary material for: MSIonization: A Machine Learning Tool for Ionization Mode Prediction of Small Molecules
Source: Anal Chem. 2026 Sep 16;98(38):27976–85. doi: 10.1021/acs.analchem.6c01937 (PMC13629163; doi:10.1021/acs.analchem.6c01937)
Supplement: Supplementary file 1 [file ac6c01937_si_001.pdf]

MSIonization: A machine learning tool for ionization mode prediction of small molecules

Yatendra Singh[1,#,&], Bakhtyar Sepehri[2,#], Zeyad Ibrahim[1], Robert J. Doerksen[2*], Sixue Chen[1*]

[1]Department of Biology, University of Mississippi, Oxford, MS 38677, USA
[2]Department of BioMolecular Sciences, University of Mississippi, Oxford, MS 38677, USA

[#]Authors contributed equally.
&Current address: Department of Pharmacology, University of Virginia, Charlottesville, VA 22908, USA

[*]**Correspondence Contact**:

Prof. Sixue Chen
Department of Biology, University of Mississippi, Oxford, MS, USA
Email: schen8@olemiss.edu

Prof. Robert J. Doerksen
Department of BioMolecular Sciences, University of Mississippi, Oxford, MS, USA
Email: rjd@olemiss.edu

Supporting information

Contents:

Figure S1. Analysis of the chemical diversity of the dataset used to develop the machine learning models: (A) Principal components PC1 (score 1) versus PC2 (score 2), (B) Tanimoto similarity heatmap, (C) Distribution of Tanimoto similarities, (D) Levenshtein distance heatmap, (E) Levenshtein similarity heatmap, and (F) Distribution of Levenshtein distances.

Figure S2. (A) Three-dimensional plot of the distribution of the number of carbon, nitrogen, and oxygen atoms in the dataset used for building ML models. (B) Two-dimensional plot of the distribution of molecular weight and logP in the dataset used for building ML models.

Figure S3. For the NPAtlas database: (A) Distribution of Tanimoto similarities, (B) Distribution of all Levenshtein distances, (C) Distribution of all Levenshtein distances less than 150.

Figure S4. For the Norman SusDat database: (A) Distribution of Tanimoto similarities, (B) Distribution of all Levenshtein distances, (C) Distribution of all Levenshtein distances less than 150.

S1. Interpretation of input variables in the C-SVM model.

Figure S5. (A) Beeswarm plot of the 19 most important MACCS fingerprints in the developed C-SVM model using the balanced training set. (B) Bar plot of the 19 most important MACCS fingerprints in the developed C-SVM model using the balanced training set.

Figure S6. Waterfall plots for molecules (A) M1, (B) M35, (C) M371, and (D) M2188 in Table S3.

S2. Tutorial for installing and running the MSIonization package.

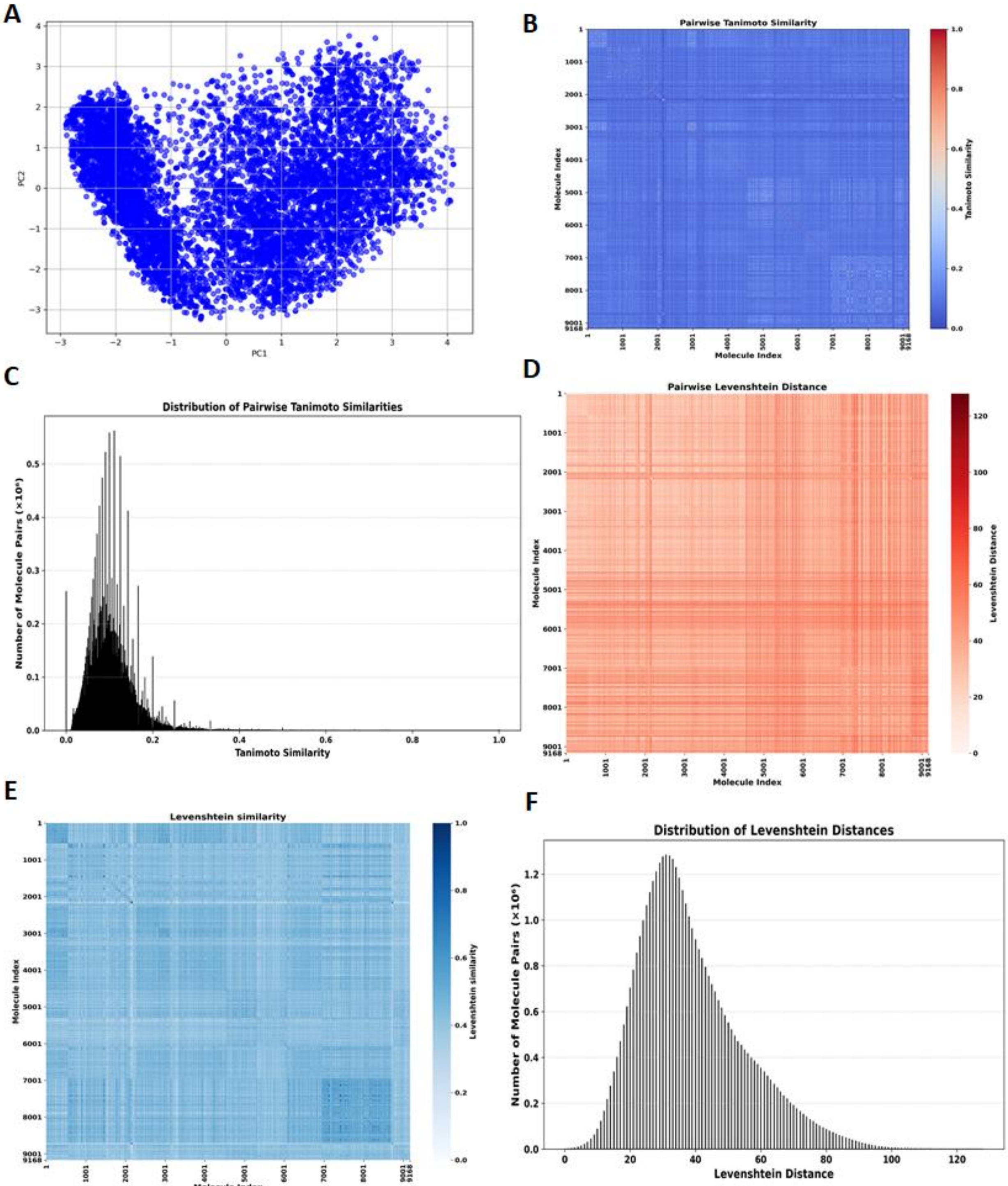

Figure S1. Analysis of the chemical diversity of the dataset used to develop the machine learning models: (**A**) Principal components PC1 (score 1) versus PC2 (score 2), (**B**) Tanimoto similarity heatmap, (**C**) Distribution of Tanimoto similarities, (**D**) Levenshtein distance heatmap, (**E**) Levenshtein similarity heatmap, and (**F**) Distribution of Levenshtein distances.

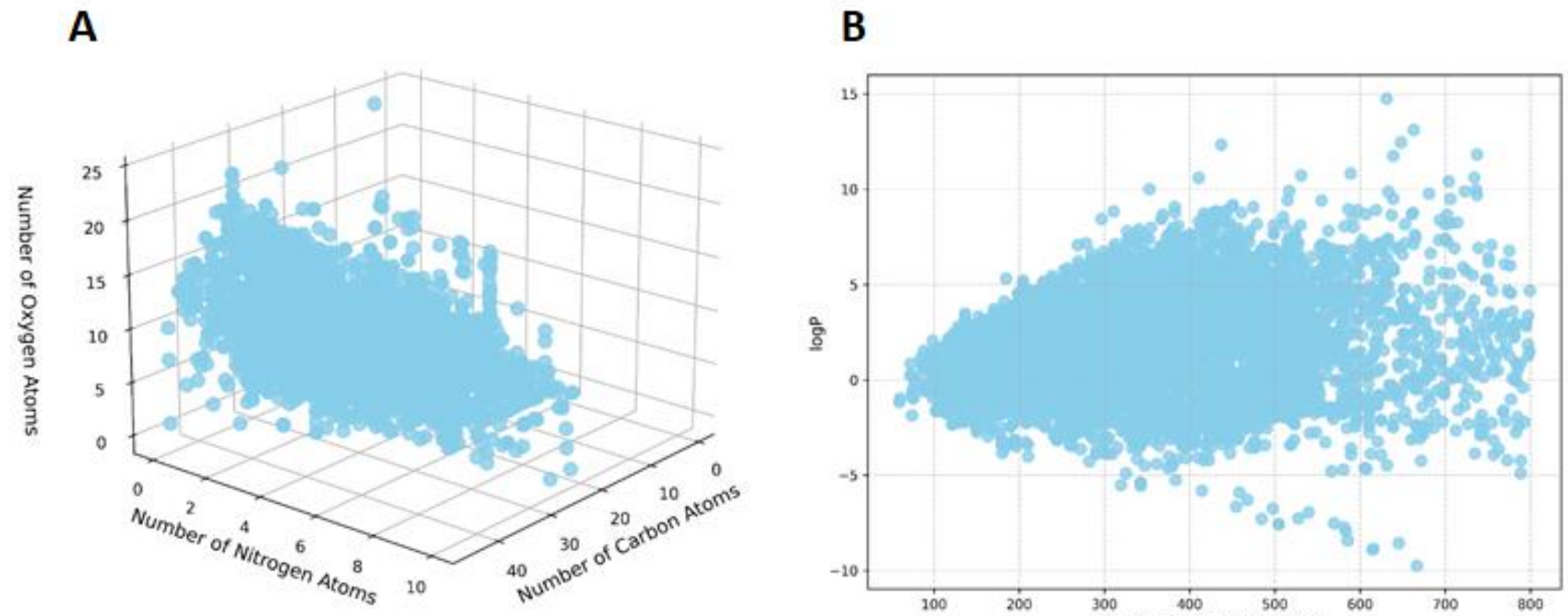

Figure S2. (**A**) Three-dimensional plot of the distribution of the number of carbon, nitrogen, and oxygen atoms in the dataset used for building ML models. (**B**) Two-dimensional plot of the distribution of molecular weight and logP in the dataset used for building ML models.

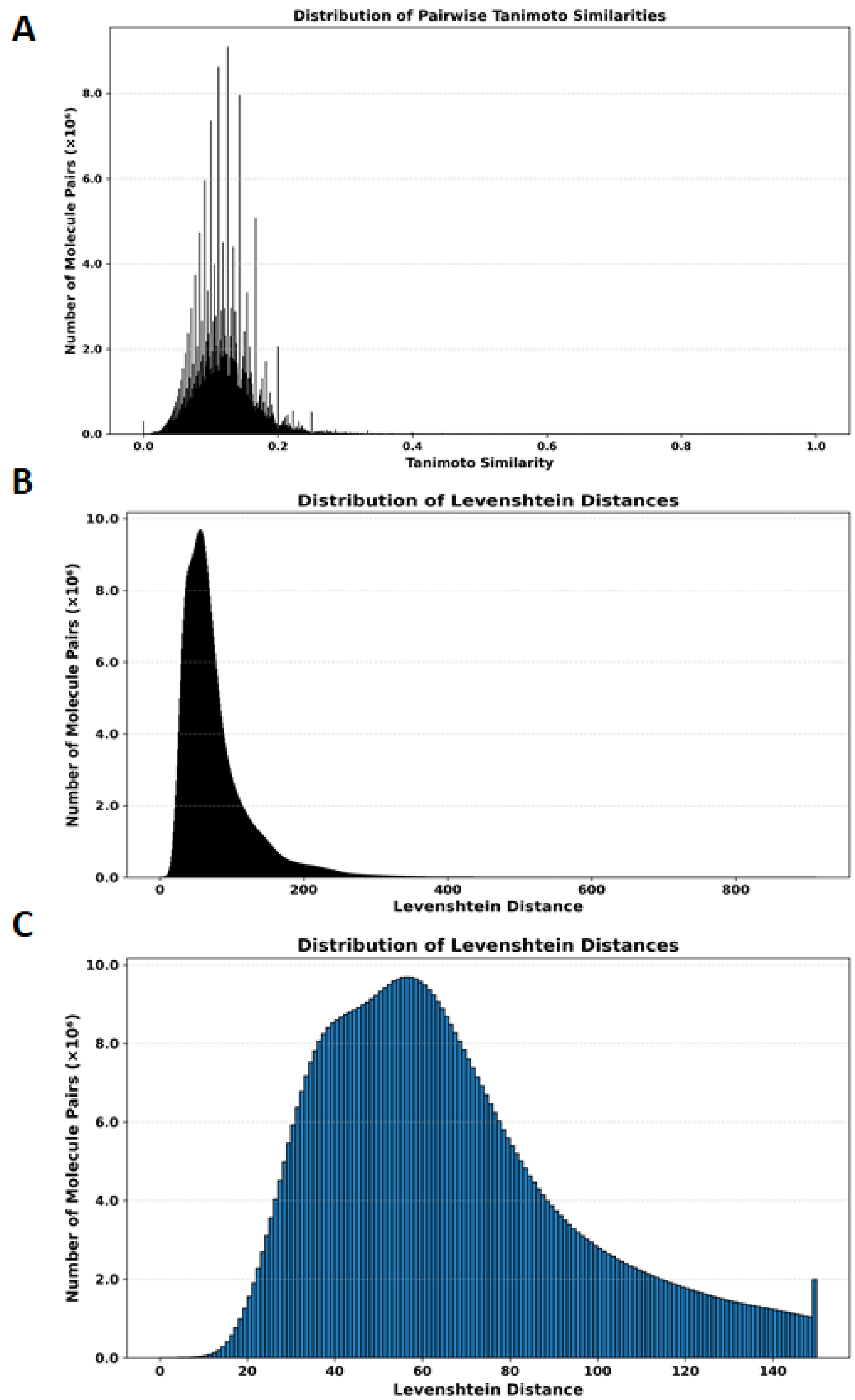

Figure S3. For the NPAtlas database: (**A**) Distribution of Tanimoto similarities, (**B**) Distribution of all Levenshtein distances, (**C**) Distribution of all Levenshtein distances less than 150.

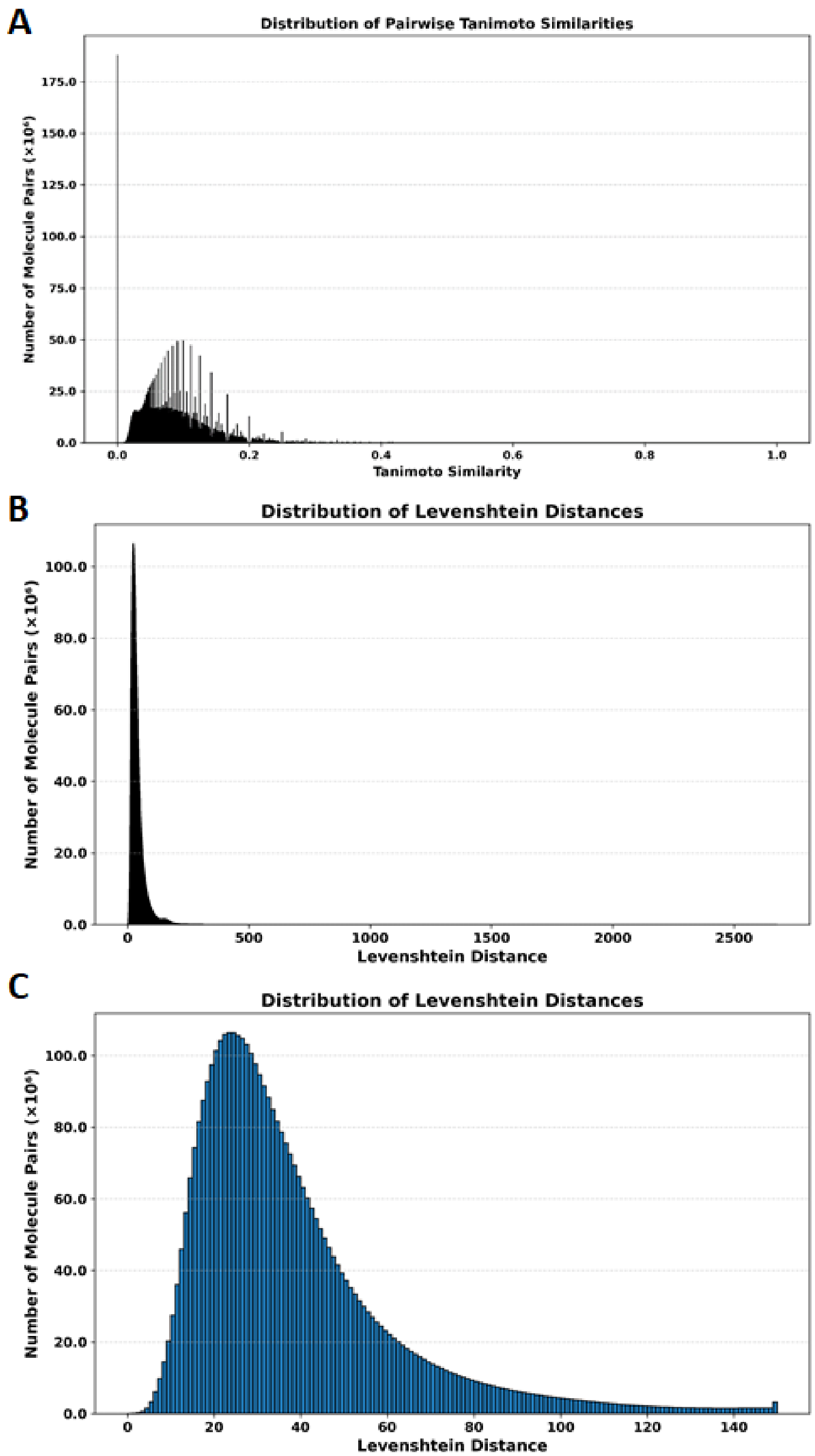

Figure S4. For the Norman SusDat database: (**A**) Distribution of Tanimoto similarities, (**B**) Distribution of all Levenshtein distances, (**C**) Distribution of all Levenshtein distances less than 150.

S1. Interpretation of input variables in the C-SVM model

We used SHapley Additive exPlanations, implemented in the SHAP software package, to determine feature importance in the developed C-SVM model using the balanced training set. Since there are many molecules in the training set, we did not use the entirety of the training set as background data. Instead, to select a representative background dataset for the balanced training set, we applied the KMeans clustering method in the scikit-learn package and did clustering, separately, on the molecules belonging to Class 0 and Class 1 in the balanced training set. The number of clusters was set to 200 for each class of molecules. and a representative molecule was selected from each cluster. This approach produced a background dataset containing 400 molecules including 200 molecules with a higher ionization efficiency in negative mode and 200 molecules with a higher ionization efficiency in positive mode. We used the Kernel SHAP method (shap.KernelExplainer) to evaluate the importance of the input variables. The explainer was built using the SVM probability prediction function (predict_proba) and the created background dataset. The link function was set to identity (link="identity"), allowing the SHAP values to sum directly to the model output probability without any transformation. In the 'explainer.shap_values' function, we set the nsamples parameter to 100. Thus, for each molecule, the model was reevaluated 100 times during the approximation of Shapley values, providing a computationally efficient estimate of feature contributions.

Figures 5A and 5B illustrate beeswarm and bar plots for the 19 most informative MACCS fingerprints. The beeswarm and bar plots show that Bit 139 is an important input variable for classifying samples, since its presence decreases the predicted probability to below the base value, whereas its absence increases it. This indicates the presence of this bit pushes the prediction toward Class 0 (molecules with higher ionization efficiency in negative mode), while its absence toward Class 1 (molecules with higher ionization efficiency in positive mode)). This bit corresponds to the presence of an oxygen atom bonded to a hydrogen atom, as found in functional groups such as hydroxyl and carboxylic acid (https://github.com/rdkit/rdkit/blob/master/rdkit/Chem/MACCSkeys.py). For molecules M4 and M9 in Table S3, this bit has a value of 1 due to the presence of a hydroxyl in M4 and a carboxylic acid in M9. Since all 166 MACCS keys fingerprint bits are binary, the higher value (red) in the beeswarm plot shows the presence of each bit, whereas the lower value (blue) shows the absence of a bit. This demonstrates the presence of carboxylic acids and hydroxyls promote a higher ionization efficiency in the negative mode, while their absence shifts classification toward Class 1. In general, molecules containing carboxylic acids or highly polar molecules with multiple hydroxyls such as glycans and phenolic compounds tend to exhibit a higher ionization efficiency in the negative mode. A similar interpretation applies to Bits 140, 123, 53, 136, 131, 54, 146, 113, 91, and 92. Bit 140 identifies molecules with more than 3 oxygen atoms, and Bit 123 is for the O-C-O motif commonly found in carboxylic acids and esters. Bit 53 is for the QHAAAQH motif, in which A is any atom in the periodic table and Q is a heteroatom (an atom other than hydrogen and carbon).

Bit 136 captures any molecule with multiple O=A motifs, representing the presence of highly polar functional groups such as carbonyl, sulfoxide, sulfate, and phosphate. Bit 131 shows the presence of more than one QH motif. Molecules which match that are likely to be highly polar molecules containing several hydroxyls. Bit 54 represents the QHAAQH motif, describing two heteroatoms bonded to hydrogen separated by two other atoms. For example, molecule M34 in Table S3 has a value of 1 for this bit because of the presence of hydroxyl groups on two adjacent carbon atoms. Bit 146 has a value of 1 for molecules containing more than two oxygen atoms and favors Class 0. Bit 113 represents the presence of an oxygen connected into an aromatic environment with a non-aromatic bond. For example, the value of this bit is 1 for molecule M23 in Table S3, which has three ethers attached to two phenyl rings. Bits 91 and 92 show the presence of $QHAAACH_2A$ and OC(N)C fragments, respectively.

As shown in the beeswarm plot (Figure S5A), Bits 161, 126, 158, 111, 138, 156, 137, and 148 shift the predicted probability above the base probability and cause the predicted class for a molecule to be Class 1. Bit 161 represents the presence of a nitrogen atom, indicating functional groups such as amines that cause molecules to have a higher ionization efficiency in the positive mode. Bit 126 matches an oxygen atom that is connected to two other atoms via non-ring (acyclic) bonds. This bit for molecule M6 is 1 due to the presence of an ether which is not a part of a ring. The presence of functional groups such as ether is associated with a molecule with higher ionization efficiency in the positive mode. Bit 158 represents a C-N fragment and captures the presence of primary, secondary, and tertiary amines, amides, and nitros, for example molecule M1928 (which contains 4-nitrobenzoic acid) in Table S3. Bits 111, 156, and 148 correspond to the presence of $NACH_2A$, NA(A)A, and AQ(A)A motifs, respectively, capturing the presence of amines. Bit 138 considers the presence of more than one QCH_2A motif. This can consider heteroatoms such as N, O, S or a halogen that is connected to a central carbon which itself is connected to any other two atoms. For example, the value of this bit for molecule M2 in Table S3 is 1 because it contains a nitrogen atom bonded to two CH_2-CH_2 fragments in the six-membered ring. Bit 137 shows the presence of heteroatoms such as N, O, and S in a ring system in a molecule. In the bar and beeswarm plots, we have not shown SHAP values for all bits since it would make both the plots overcrowded.

The ionization efficiency is influenced by many structural features and since the dataset we used is highly diverse, a limited number of fingerprints cannot explain completely the classification of molecules. The situation is more complex when factors beyond molecular structure can also affect the ionization process. Figure S6A shows waterfall plots for molecules M1 ((+)-chloropseudoephedrine), M35 (3',4'-dihydroxyphenylacetone), M371 (3-CAF), and M2188 (taurine). Molecule M1 has an amine group and is able to be ionized only in the positive mode. As shown in Table S3, the predicted class by the C-SVM model for this molecule is 1. Figure S6A shows that the absence of a carboxylic acid (Bit 139), the QO motif (Bit 102), ON(C)C motif (Bit 13) and Bit 135 (a nitrogen atom connected by

a non-aromatic bond to an atom, which is then connected by an aromatic bond to another atom) have pushed the predicted probability above the base value and caused the predicted class to be Class 1 for this molecule. Data in Table S3 show molecule M35 has a high ionization efficiency in both modes but its higher ionization efficiency in the negative mode caused it to be assigned to Class 0. The C-SVM model correctly predicted this class. In its chemical structure, there are two hydroxyls and a ketone. Figure S6B shows the waterfall plot for this molecule. As illustrated in this figure, the presence of hydroxyls (Bit 139, OH motif), an oxygen attached to other atoms through non-aromatic bonds (a ketone not attached to a phenyl ring in this molecule), Bit 113 and Bit 54 (QHAAQH motif, showing the presence of two hydroxyl groups on adjacent carbons in the phenyl ring) and the absence of Bit 8 (4-membered ring containing at least one heteroatom), Bit 93 (QCH_3 motif), Bit 100 (ACH_2N motif), Bit 120 (more than one heteroatom inside its ring systems), bit 161 (the presence of a nitrogen atom) and Bit 11 (4-membered ring) shift the predicted probability below the base value and cause the predicted class for this molecule be Class 0. For molecule M371, which ionizes only in the positive mode, the presence of Bit 94 (the presence of the QN fragment, which is N-N in molecule M371) and Bit 163 (6-membered ring), together with the absence of Bit 139 (OH group), Bit 66 (a carbon bound to four other atoms among which three of them are carbons), Bit 128 (ACH2AAACH2A motif), Bit 88 (the presence of sulfur), Bit 69 (QQH motif), Bit 146 (more than two oxygen atoms), and Bit 74 (CH_3ACH_3 motif) increased the predicted probability above the base value and led to classification as Class 1 (Figure S6C). Molecule M2188 (taurine, 2-aminoethanesulfonic acid) can be ionized in both modes but has a higher ionization efficiency in the negative mode and was therefore assigned to Class 0. Figure S6D shows that the presence of Bit 61 (AS(A)A, capturing the sulfonic acid in this molecule) and Bit 139 (the OH group, as well as the OH group in the sulfonic acid) and the absence of Bit 115 (CH_3ACH_2A), Bit 145 (more than one 6-membered ring), Bit 59 (sulfur atom that is not part of an aromatic two-bond sequence), Bit 28 (QCH_2Q motif), Bit 38 (NC(C)N motif), and Bit 72 (OAAO motif) shifted the prediction toward Class 0.

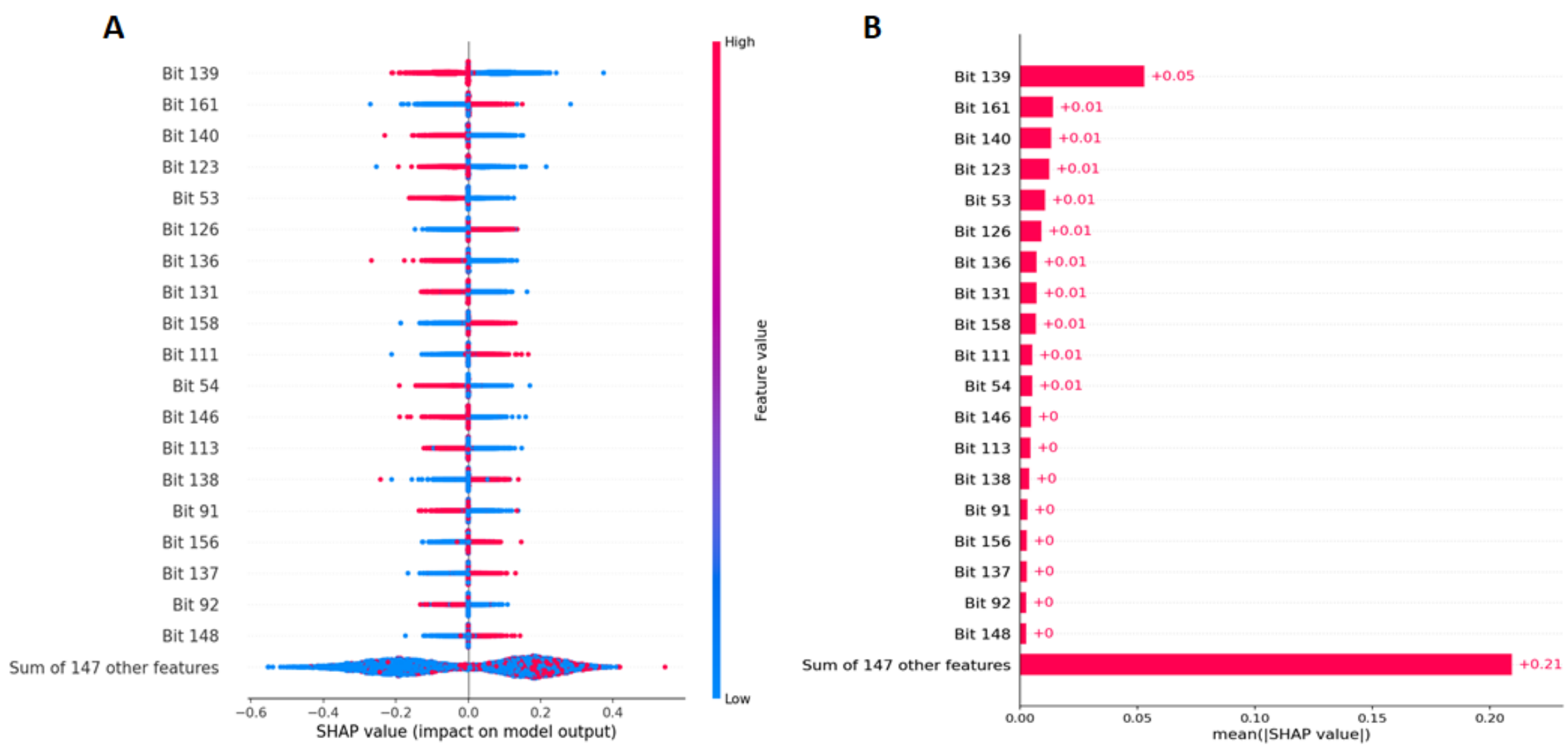

Figure S5. **(A)** Beeswarm plot of the 19 most important MACCS fingerprints in the developed C-SVM model using the balanced training set. (**B**) Bar plot of the 19 most important MACCS fingerprints in the developed C-SVM model using the balanced training set.

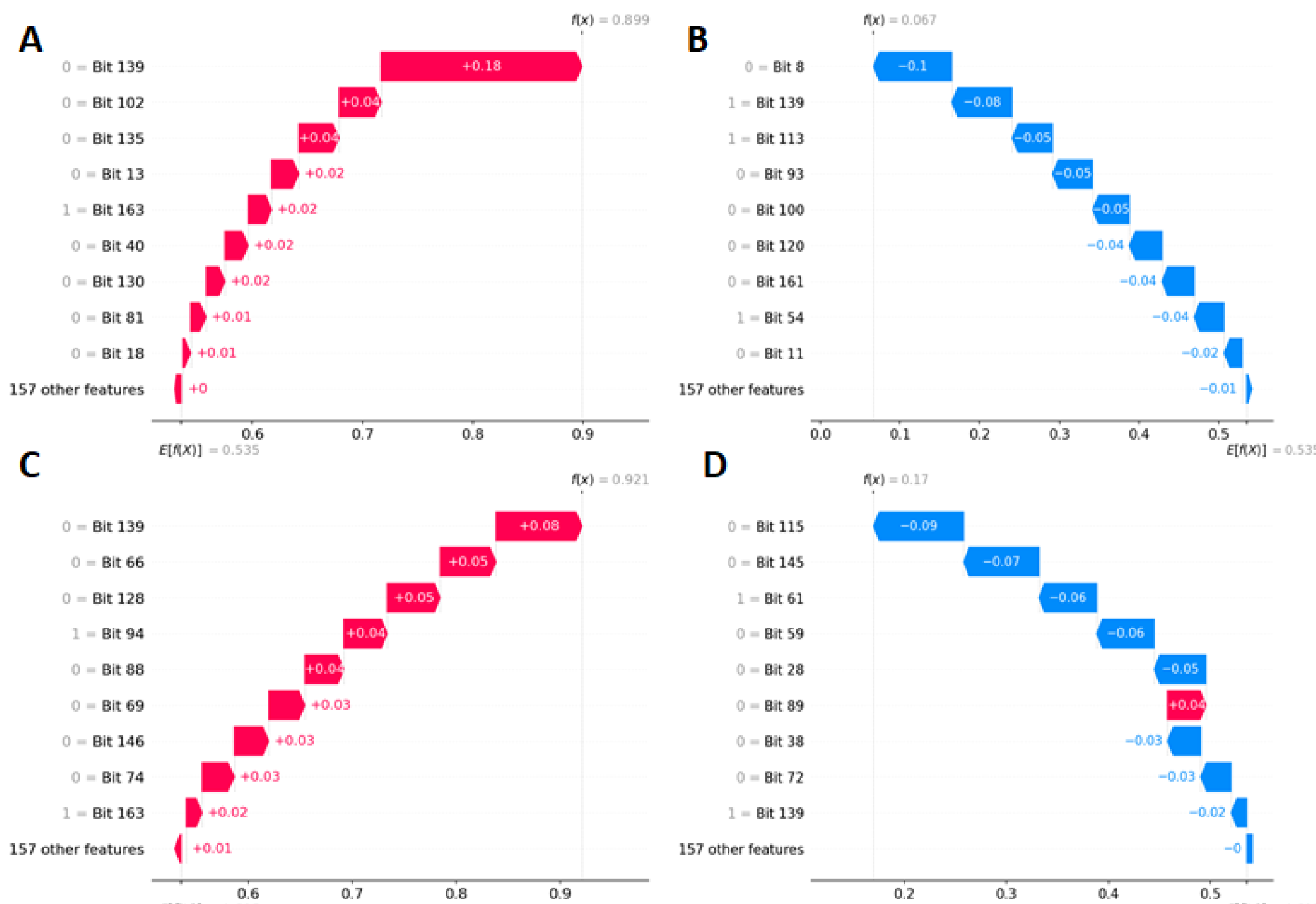

Figure S6. Waterfall plots for molecules: (A) M1, (B) M35, (C) M371, and (D) M2188 in Table S3.

S2. Tutorial for installing and running the MSIonization package

1. Installing the MSIonization package

1) Download and install the latest version of Python for the macOS or Windows operating system from this link: https://www.python.org/
2) Check the Python version by typing this command in the terminal:
   macOS/Linux: python3 -version
   Windows: py -version
3) For installing the MSIonization package, the pip package must be installed on your computer. The user can check if it is installed by the following commands:
   Windows: py -m pip -version
   Linux/macOS: python3 -m pip -version
4) If pip has not been installed, the user can install it by using:
   Linux/macOS: python3 -m ensurepip -default-pip
   Windows: py -m ensurepip --default-pip
5) If it still has not been installed, the user can download the get-pip.py script from this link: https://bootstrap.pypa.io/get-pip.py
   Go to the downloaded folder (directory) in cmd (in Windows) or Terminal (in Linux or macOS) and then run this command: python get-pip.py
6) The user can navigate to the folder (or directory) containing the MSIonization package in cmd (in Windows) or Terminal (in Linux or macOS) and install the package using this command:
   macOS/Linux: pip install MSIonization-1.0-py3-none-any.whl
   Windows: py -m pip install MSIonization-1.0-py3-none-any.whl
7) The computer must have an internet connection to install the required packages (numpy, scikit-learn, rdkit, joblib, and pandas) for using the MSIonization package. These required packages will be installed automatically.

2. Verifying the installation

1) Open the terminal in macOS/Linux or cmd in Windows and run the appropriate command:
   macOS/Linux: pip show MSIonization
   Windows: py -m pip show MSIonization

3. Running MSIonization Using a Python Script or Spyder IDE

1) Install Spyder IDE using:
   macOS/Linux: pip install spyder (internet connection is required).
   Windows: py -m pip install spyder (internet connection is required).
2) To make a prediction, put the code from steps 4-6 into a Python script and run it from the terminal using: python3 script_name.py
3) To run code using Spyder IDE, load it in the terminal by typing: spyder

4. Making Predictions

Prediction for a single molecule:

```
#################################################################
from MSIonization import predict_ionization
smiles = "CC(C(C1=CC=CC=C1)Cl)NC"
result = predict_ionization(smiles)
print(result)
#################################################################
```

In this code, the user can change the SMILES notation to whatever is needed.

4) Prediction for multiple molecules:

```
#################################################################
from MSIonization import predict_ionization
# List of multiple SMILES strings
smiles_list = ["CC(C(C1=CC=CC=C1)Cl)NC",
"CC12CCC3C(C1CCC2OC4C(C(C(C(O4)C(=O)O)O)O)O)CCC5=CC(=O)CCC
35", "C1CN1P(=O)(N2CC2)N3CC3",
"CC(COCC(C)OC(=O)C1=CC=CC=C1)OC(=O)C2=CC=CC=C2"]
# Prediction for all SMILES
for smiles in smiles_list:
    result = predict_ionization(smiles)
    print(f"SMILES: {smiles} -> Ionization: {result}")
#################################################################
```

5) Batch prediction for a list of SMILES strings from a CSV (a column with 'SMILES' header is required):

```
#################################################################
import pandas as pd
from MSIonization import predict_ionization

# Read SMILES strings from a CSV file
data = pd.read_csv("SMILES.csv")

# Ensure that the CSV file contains a column with the header 'SMILES.'
if 'SMILES' not in data.columns:
    raise ValueError("CSV file must contain a 'SMILES' column.")

# Running prediction for all SMILES in CSV file
for smiles in data['SMILES'].dropna():
    try:
        result = predict_ionization(smiles)
        print(f"SMILES: {smiles} -> Ionization: {result}")
    except Exception as e:
        print(f"Error processing SMILES '{smiles}': {e}")
#################################################################
```

Keynote 1: The CSV file must be put in the working directory of spyder IDE.

Keynote 2: All three of these codes can be found in the 'MSIonization_commands.py' file.

5. Running the MSIonization package using the MSIonization_script.py file

1) To make predictions for a list of SMILES strings in a CSV file and save the results in another CSV file, we have provided a separate script called 'MSIonization_script.py'. The user can put both the script and the CSV file in the same directory. Then, open the terminal (macOS/Linux) or cmd (in Windows) in the same directory and run the following command: python3 MSIonization_script.py

6. Running the MSIonization package using the GUI

1) For loading the designed GUI, the user needs to install the tkinter and setuptools packages:
   To install setuptools:
   macOS/Linux: pip install setuptools
   Windows: py -m pip install setuptools
   To install tkinter on different operating systems:
   Rocky Linux (RHEL-based): sudo dnf install python3-tkinter
   Ubuntu Linux (Debian-based): sudo apt install python3-tk
   macOS: brew install python-tk
   Windows: Tkinter is usually included with the standard Python installation.
2) Install the MSIonization package as described above in the 'Installing the MSIonization package' section.
3) Load GUI on macOS/Linux by typing this command and pressing the enter key: MSIonization-GUI
4) To load the GUI in Windows, navigate to the installation directory in cmd by using this command: cd Installation directory
   Example:
   cd C:\Users\lab\AppData\Local\Programs\Python\Python313\Scripts\
   Then load the GUI by this command in cmd: MSIonization-GUI.exe
5) This GUI consists of two panels for importing molecules, running predictions, and finally saving results. In the first panel (Option 1: Paste SMILES), there is a place to paste a list of SMILES notations for any number of molecules. After providing the SMILES notation of molecules, the user needs to click the 'Predict' button and wait until all calculations are completed. During this step, a message in blue will appear that shows calculations are running. After completing the prediction step, a message in green will appear showing that the prediction step has been completed. Then the results could be saved in a CSV file using the 'Save Results' button.

The second panel is the 'Option 2: Import SMILES CSV' panel. In this panel, the user can import a list of SMILES notations in a CSV file named 'SMILES.csv' that contains one column with the header 'SMILES'. To do this, the user can click on 'Import CSV' to select and import the file. The user can then run the prediction using the 'Predict' button and save the results using the 'Save Results' button. The CSV output file (Predicted_Ionization_efficiencies.csv) contains four columns, including SMILES, Predicted class, Probability, and Applicability domain.

7. Installing the MSIonization package using Anaconda

1) Download Anaconda from: https://www.anaconda.com/download/success
2) Anaconda installation guide: https://www.anaconda.com/docs/getting-started/anaconda/install#macos-linux-installation
3) Anaconda includes pip, Spyder IDE, and package management tools.
4) Open **Terminal** (macOS/Linux) or **Anaconda Prompt** (Windows).
5) Navigate to the folder containing the MSIonization .whl file and install:

pip install MSIonization-1.0-py3-none-any.whl

6) Launch the GUI: MSIonization-GUI

8. Keynote: To open cmd in a directory or a folder in Windows, follow these instructions:

1) Navigate to the desired folder in File Explorer.
2) Click on the address bar (or press Alt + D) to highlight it.
3) Type cmd and press Enter.
